# Effects of seasonal and pandemic influenza on health‐related quality of life, work and school absence in England: Results from the Flu Watch cohort study

**DOI:** 10.1111/irv.12506

**Published:** 2018-02-19

**Authors:** Ellen B. Fragaszy, Charlotte Warren‐Gash, Peter J. White, Maria Zambon, William J. Edmunds, Jonathan S. Nguyen‐Van‐Tam, Andrew C. Hayward, on behalf of the Flu Watch Group

**Affiliations:** ^1^ Institute of Health Informatics University College London London UK; ^2^ Faculty of Epidemiology & Population Health London School of Hygiene & Tropical Medicine London UK; ^3^ MRC Centre for Outbreak Analysis and Modelling and NIHR Health Protection Research Unit in Modelling Methodology Imperial College London School of Public Health London UK; ^4^ Modelling and Economics Unit National Infection Service Public Health England London UK; ^5^ National Infection Service Public Health England London UK; ^6^ Health Protection and Influenza Research Group Division of Epidemiology and Public Health University of Nottingham Nottingham UK; ^7^ Institute of Epidemiology & Healthcare University College London London UK

**Keywords:** costs and cost analysis, EQ‐5D, human, influenza, quality of life, respiratory tract infections, work and school absences

## Abstract

**Background:**

Estimates of health‐related quality of life (HRQoL) and work/school absences for influenza are typically based on medically attended cases or those meeting influenza‐like‐illness (ILI) case definitions and thus biased towards severe disease. Although community influenza cases are more common, estimates of their effects on HRQoL and absences are limited.

**Objectives:**

To measure quality‐adjusted life days and years (QALDs and QALYs) lost and work/school absences among community cases of acute respiratory infections (ARI), ILI and influenza A and B and to estimate community burden of QALY loss and absences from influenza.

**Patients/methods:**

Flu Watch was a community cohort in England from 2006 to 2011. Participants were followed up weekly. During respiratory illness, they prospectively recorded daily symptoms, work/school absences and EQ‐5D‐3L data and submitted nasal swabs for RT‐PCR influenza testing.

**Results:**

Average QALD lost was 0.26, 0.93, 1.61 and 1.84 for ARI, ILI, H1N1pdm09 and influenza B cases, respectively. 40% of influenza A cases and 24% of influenza B cases took time off work/school with an average duration of 3.6 and 2.4 days, respectively. In England, community influenza cases lost 24 300 QALYs in 2010/11 and had an estimated 2.9 million absences per season based on data from 2006/07 to 2009/10.

**Conclusions:**

Our QALDs and QALYs lost and work and school absence estimates are lower than previous estimates because we focus on community cases, most of which are mild, may not meet ILI definitions and do not result in healthcare consultations. Nevertheless, they contribute a substantial loss of HRQoL on a population level.

## 1 INTRODUCTION

Influenza epidemics have a major social and economic impact. As well as direct healthcare costs, influenza may lead to other indirect effects including school absenteeism, loss of workplace productivity and effects on health‐related quality of life (HRQoL).^1^ The quality of life of both patients and their families may be affected, especially when the patient is a child.^2^ Quantifying indirect effects accurately is essential to inform cost utility analyses (CUA) of interventions to mitigate the population impact of influenza, including extension of seasonal vaccination policies.

In the United Kingdom, the National Institute for Health and Care Excellence (NICE) recommends that health effects of interventions are expressed in terms of quality‐adjusted life years (QALYs) as this generic measure of health benefits reflect both mortality and HRQoL.^3^ The standardised validated tool EQ‐5D^4^ is NICE's preferred measure of HRQoL.^3^ NICE uses a cost utility threshold of £20 000‐30 000 per QALY to judge whether or not interventions are deemed cost effective.

A systematic review of HRQoL in influenza showed a paucity of studies that used standardised well‐validated methods to generate estimates of the quality‐adjusted life days (QALDs) lost.^5^ It identified 4 previous estimates of QALDs lost due to influenza, which varied from 1.57 to 10.69 depending on the population sampled and method of HRQoL measurement used.^6^, ^7^, ^8^, ^9^ Many of these studies did not measure HRQoL throughout the duration of illness. They tended to measure HRQoL once at baseline and once on the worst day of illness, which required assumptions to be made about the shape of the QALY loss over an illness.^5^

Studies that measure HRQoL and work and school absence from influenza cases seeking medical attention may overestimate the indirect cost per case. A systematic review of studies of children's absences from school and day care due to influenza showed a gradient of days lost, with the longest absences reported by cases attending hospital emergency departments, then those in physician office‐based studies followed by community cases.^10^ Additionally, studies that estimate the population‐level burden of HRQoL and absences from only severe cases miss the majority of influenza illnesses which, despite their mild nature, are likely to contribute substantially to the overall burden.^5^, ^11^ Although household studies may capture these milder illnesses that do not result in health‐seeking behaviour, and therefore provide less biased estimates, their specificity is often limited by a lack of laboratory‐confirmed diagnoses.

There is therefore a need for robust estimates of the indirect effects of influenza from community studies identifying illnesses through prospective active symptom and molecular surveillance. We have previously described the community burden of influenza, ILI and acute respiratory infections not meeting the definition of ILI across multiple influenza seasons in a large household cohort in England.^12^ Here, we present the effects of these illnesses on HRQoL and work/school absences using the same cohort. We also estimate the population‐level burden of these outcomes among community influenza cases.

## 2 METHODS

### 2.1 Study design

Flu Watch is a previously described, household‐based, community cohort study of acute respiratory disease and influenza infection in England.^12^, ^13^ In brief, the study followed up cohorts during 6 influenza seasons including 3 periods of seasonal influenza (winters 2006‐2007, 2007‐2008 and 2008‐2009) and the first 3 waves of the 2009 influenza pandemic (summer 2009, autumn‐winter 2009/2010 and winter 2010/2011). In total, 5484 participants were followed up for 118 158 person‐weeks. Individuals were randomly recruited through primary care practices and their households invited to participate. Participants gave written informed consent, and parents/guardians gave proxy consent for children. The Flu Watch study was approved by the Oxford MultiCentre Research Ethics committee (06/Q1604/103).

Baseline surveys collected demographic, socio‐economic and occupation data. Participants were categorised into “working” (employed full‐time, part‐time or self‐employed), “students” (self‐classified, aged 5‐15) and “not in work/education.” Participants were contacted weekly and asked to record any “cough, cold, sore throat or flu‐like illness”, which we define as an acute respiratory illness. During these illnesses, participants reported daily symptoms and temperature measurements using prospective illness diaries. Parents/guardians completed surveys on behalf of their children as needed. Self‐administered nasal swabs were requested on day 2 of any illness. Participants submitted the swabs by mail to be tested for circulating influenza A viruses (H1N1, H3N2 and from 2009 onwards H1N1pdm09) and influenza B viruses using RT‐PCR.^14^, ^15^

### 2.2 HRQoL outcomes

Between 2006/2007 and 2009/2010 illness diaries included daily questions on whether the ill individual had taken time off work/school. In 2006/2007 through 2008/2009 and for a subset of participants in 2009/2010, illness diaries also asked whether someone else took time off on that day to care for them. During 2009/2010, time off was quantified as ≤4 or >4 hours. In 2010/2011, QALDs and QALYs were measured using the EQ‐5D‐3L instrument,^16^, ^17^, ^18^ which was completed at baseline and daily throughout illness. Designed for self‐completion, EQ‐5D‐3L has 2 components. The first describes health across 5 domains: mobility, self‐care, usual activities, pain and anxiety. Participants rate each domain as “no problems,” “some problems” or “extreme problems.” Participants also record their overall health status on a visual analogue scale (EQ‐VAS) from 0 (worst imaginable health state) to 100 (best imaginable health state). The online EQ‐VAS question used in Flu Watch however asked participants to rate their health without the visual scale. The 3 possible responses for each of the 5 EQ‐5D‐3L domains results in 3^5^ possible health states. These health states were mapped to an index value (representing a QALD weight) using a validated UK value set.^18^ The QALD weights range between 1 (full health) and 0 (dead).

### 2.3 Illness outcomes

All acute respiratory illnesses, regardless of swabbing or PCR result, were classified into 2 symptomatic outcomes. Those with confirmed fever (≥37.8°C) or symptoms of “feeling feverish” and either a cough or sore throat at any point were classified as influenza‐like illnesses (ILI). All other acute respiratory illnesses were classified as acute respiratory infections (ARI). Among the illnesses that had an accompanying swab, some were confirmed as PCR+ influenza cases and these were grouped into influenza A and influenza B viruses. In 2010/2011, when the EQ‐5D‐3L data were collected, all influenza A illnesses were H1N1pdm09, apart from 1 H3N2 case. The individual‐level results report QALD loss for H1N1pdm09 cases only, but the population projections include H3N2.

### 2.4 Statistical analysis

#### 2.4.1 Time off work/education

The illness duration, percentages of illnesses with time off and mean number of days taken off were calculated for each illness outcome and stratified by age group and employment status. The latter 2 estimates were carried out separately for time off taken by the ill person, by someone caring for the ill person and a combination of both.

#### 2.4.2 HRQoL

Within each illness, the worst day of illness within each domain was identified. The percentage of respondents reporting no, some or extreme problems on their worst day in each domain was compared to the corresponding baseline responses, stratified by illness outcome.

Within each illness, the worst day for EQ‐VAS and the worst day for QALD weight were identified. For each illness outcome, mean and median worst day EQ‐VAS scores and QALD weights were calculated and compared to baseline measurements.

Total QALD loss for each illness was calculated by subtracting the daily QALD weights taken during illness from the participant's baseline QALD weight and summing these differences up over the course of the illness. Mean and median total QALD and QALY losses per illness were calculated by illness outcome and stratified by age group and whether or not cases were medically attended.

A sensitivity analysis was also conducted using the respondents' highest reported QALD weight as the comparison (baseline) group, regardless of when it was measured.

#### 2.4.3 Missing data

If a participant's baseline questionnaire was missing, then QALDs and QALYs could not be estimated for their subsequent illnesses. All illnesses with daily EQ‐5D‐3L measurements were included in the duration of illness, worst day EQ‐VAS and QALD weight estimates. If a participant failed to complete illness diaries throughout their illness, then their illness duration would be truncated. We also investigated whether influenza cases actively reported no illness in the week following the last reported day of illness, or whether this weekly report was missing.

#### 2.4.4 Population impact

We estimated the total QALY loss experienced by community cases in the population and the number of days they took time off work/school due to influenza. Estimates were obtained from Flu Watch data by taking 25 000 Monte Carlo samples from the distributions of incidence of illness and QALD losses, or days off work, as appropriate, for each age group. The incidence of illness and HRQoL outcomes for the QALY analysis were derived from 2010/2011 data while estimates for the absence analysis came from 2006/2007‐2009/2010. The mid‐2011 population size and age‐distribution for England was used.^19^

## 3 RESULTS

In total, 2919 participants reported 4818 illnesses (2805 ARI and 2013 ILI; Table 1). Of the 3161 illnesses with nasal swabs, 177 tested positive for influenza A and 45 for influenza B. 75% of influenza A cases meet our ILI case definition however only 48% reported fever (a symptom required for many ILI definitions). For influenza B, 80% of cases met our ILI definition but only 60% reported fever. Most influenza B cases were in children whereas most influenza A cases were in adults. 25% of influenza A cases and 14% of influenza B cases were medically attended either through the government run pandemic influenza Web or phone service (which ran during 2009/10), the NHS Direct telephone service, or contact with a GP, accident and emergency department or hospital.

**Table 1:** Baseline characteristics of ill participants

	All people		All illnesses		All illnesses (N = 4818)				Illnesses tested for Flu A & B (N = 3161)			
					ARI		ILI		Influenza A PCR+		Influenza B PCR+	
	n	%	n	%	n	%	n	%	n	%	n	%
Overall	2919	100	4818	100	2805	100	2013	100	177	100	45	100
By influenza season												
Winter 2006/2007	270	9	399	8	146	5	253	13	14	8	0	0
Winter 2007/2008	363	12	539	11	188	7	351	17	10	6	4	9
Winter 2008/2009	219	8	410	9	123	4	287	14	40	23	13	29
Summer 2009	33	1	110	2	42	2	68	3	2	1	0	0
Winter 2009/2010	1644	56	2690	56	1893	68	797	40	75	42	5	11
Winter 2010/2011	390	13	670	14	413	15	257	13	36	20	23	51
By age group												
0‐15 y	647	22	1203	25	648	23	555	28	68	39	26	58
16‐65 y	1806	63	2892	61	1723	62	1169	59	99	57	15	33
65 y and over	431	15	679	14	409	15	270	14	8	5	4	9
By IMD quartile^a^												
1 (most deprived)	141	5	238	5	132	5	106	5	12	7	3	7
2	606	21	1032	22	544	20	488	25	49	28	12	27
3	1010	35	1715	36	1012	37	703	36	55	31	14	31
4 (least deprived)	1099	38	1750	37	1065	39	685	35	59	34	16	36
By occupation												
In work	1288	51	2052	46	1267	51	785	47	62	41	9	22
Student	533	21	932	21	510	21	422	25	59	39	25	61
Not in work/school	724	28	1172	26	708	29	464	28	30	20	7	17

	1513	53	2574	54	1491	54	1083	55	89	51	23	51
	1343	47	2161	46	1262	46	899	45	86	49	22	49

a English indices of multiple deprivations 2007.

### 3.1 Time off work/education

Average illness duration, percentages of illnesses with time off and the symptom number of days per illness with time off were broadly comparable between influenza A and B cases although influenza A appeared slightly more severe (Table 2). Illness duration was 9.6 and 10.7 days for influenza A and B, respectively. Among cases where absence data were available for both the ill participant and those caring for them, 50% of influenza A and 41% of influenza B cases required at least 1 person to take time off for a combined average of 5.0 and 3.4 days, respectively. Among ill children, 56% and 31% took time off school or childcare for an average duration of 3.5 and 2.1 days for influenza A and B, respectively. Among the subset of data where information was available, 70% and 42% of children's illnesses required someone else to take time off to care for them. Ill adults were less likely to take time off (31% and 20% for influenza A and B, respectively) but took more time off (3.8 and 3.0 days for influenza A and B, respectively). Estimates remained similar when limited to working adults aged 16 and over. ILI cases were broadly comparable with influenza cases although more severe than the ARI cases. For the 142 influenza illnesses where the amount of time taken off per day was measured, 83% of days had more than 4 hours off.

**Table 2:** Illness duration and time off work/education (Autumn 2006 – Spring 2010)

	ARI		ILI		Flu A PCR+		Flu B PCR+	
	N	Estimate	N	Estimate	N	Estimate	N	Estimate
Overall								
Duration of symptoms, average (min, max)	2805	6.9 (1, 48)	2013	9.0 (1, 82)	177	9.6 (1, 82)	45	10.7 (1, 65)
Percent of illnesses where the ill participant and/or someone caring for them takes time off work/education/childcare^a^	458	11%	897	30%	64	50%	17	41%
Among illnesses with anyone's time off: Average number of days someone (regardless of who) takes time off work/education/childcare (min, max)^a^	51	2.5 (1, 6)	269	3.8 (1, 18)	32	5.0 (2, 11)	7	3.4 (2, 6)
Percent of ill participants taking time off work/education/childcare	2805	11%	2013	27%	177	40%	45	24%
Among ill participants taking time off: Average number of days they take time off work/education/childcare (min, max)	296	2.5 (1, 14)	545	3.2 (1, 18)	71	3.6 (1, 13)	11	2.4 (1, 4)
Percent of illnesses where someone else takes time off to care for ill participant^a^	458	4%	897	11%	64	28%	17	29%
Among illnesses where someone else takes time off: Average number of days they take time off to care for ill participant (min, max)^a^	19	1.4 (1, 3)	102	2.0 (1, 7)	18	2.7 (1, 6)	5	1.6 (1, 2)
Ill Children (0‐15 y)^b^								
Percent of ill children taking time off school/childcare for their illness	648	14%	555	39%	68	56%	26	31%
Among ill children taking time off: Average number of days they take time off school/childcare (min, max)	93	2.3 (1, 12)	218	2.9 (1, 13)	38	3.5 (1, 13)	8	2.1 (1, 4)
Percent of illnesses where someone else takes time off to care for ill child^a^	78	10%	256	24%	20	70%	12	42%
Among illnesses where someone else takes time off: Average number of days they take time off to care for ill child (min, max)^a^	8	1.6 (1, 3)	61	2.2 (1, 7)	14	2.9 (1, 6)	5	1.6 (1, 2)
Ill Adults (16‐64 y)^b^								
Percent of ill adults taking time off work/education for their illness	1723	11%	1169	26%	99	31%	15	20%
Among ill adults taking time off: Average number of days they take time off work/education (min, max)	184	2.6 (1, 14)	303	3.3 (1, 18)	31	3.8 (1, 9)	3	3.0 (2, 4)
Percent of illnesses where someone else takes time off to care for ill adult^a^	319	3%	535	7%	39	10%	5	0%
Among illnesses where someone else takes time off: Average number of days they take time off to care for ill adult (min, max)^a^	11	1.2 (1, 2)	35	1.5 (1, 5)	4	2.0 (1, 3)	00	N/A
Ill Older Adults (65+ years)^b^								
Percent of ill older adults taking time off work/education for their illness	409	5%	270	9%	8	13%	4	0%
Among ill older adults taking time off: Average number of days they take time off work/education (min, max)	19	3.4 (1, 7)	23	5.3 (1, 14)	1	3.0 (3, 3)	0	N/A
Percent of illnesses where someone else takes time off to care for ill older adult^a^	61	0%	105	6%	4	0%	0	N/A
Among illnesses where someone else takes time off: Average number of days they take time off to care for ill older adult (min, max)^a^	0	N/A	6	2.5 (1, 5)	0	N/A	0	N/A
Ill Working Adults (16+ years)^b^								
Percent of ill working adults taking time off work/education for their illness	1267	12%	785	30%	62	34%	9	33%
Among ill working adults taking time off: Average number of days they take time off work/education (min, max)	155	2.6 (1, 14)	233	3.3 (1, 18)	21	4.0 (1, 9)	3	3.0 (2, 4)
Percent of illnesses where someone else takes time off to care for ill working adult^a^	235	4%	361	6%	24	13%	2	0%
Among illnesses where someone else takes time off: Average number of days they take time off to care for ill working adult (min, max)^a^	10	1.2 (1, 2)	23	1.2 (1, 3)	3	2.3 (2, 3)	0	N/A

a Estimates limited to subset of data where time off work/education information was col lected for both ill participant and anyone caring for them.

b Age group missing for 2 Influenza A cases, 7 ILI cases and 25 ARI cases.

### 3.2 EQ‐5D‐3L

**Figure 1:**
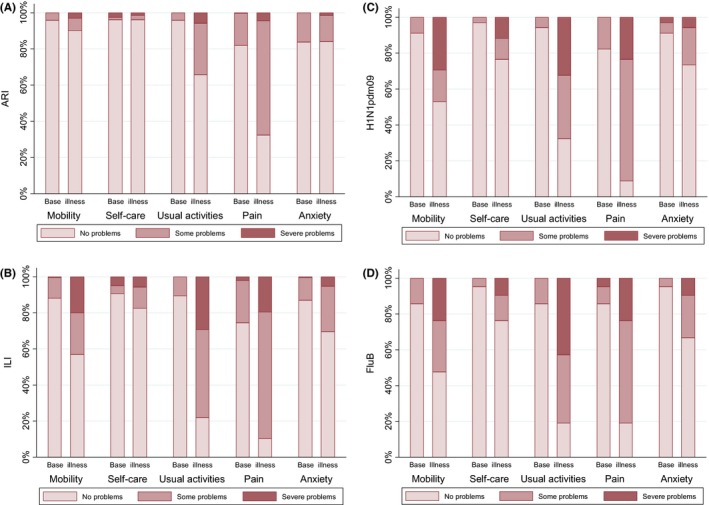
EQ‐5D‐3L domains comparing baseline and worst day of illness (for the respective domain) for (A) ARI (B) ILI, (C) H1N1pdm09 and (D) influenza B illnesses

Those reporting problems and problem severity on the worst day of illness were broadly similar between H1N1pdm09, influenza B and ILI (Figure 1 a‐d). The most affected domains were “usual activities” and pain, followed by mobility, but all domains were affected.

**Figure 2:**
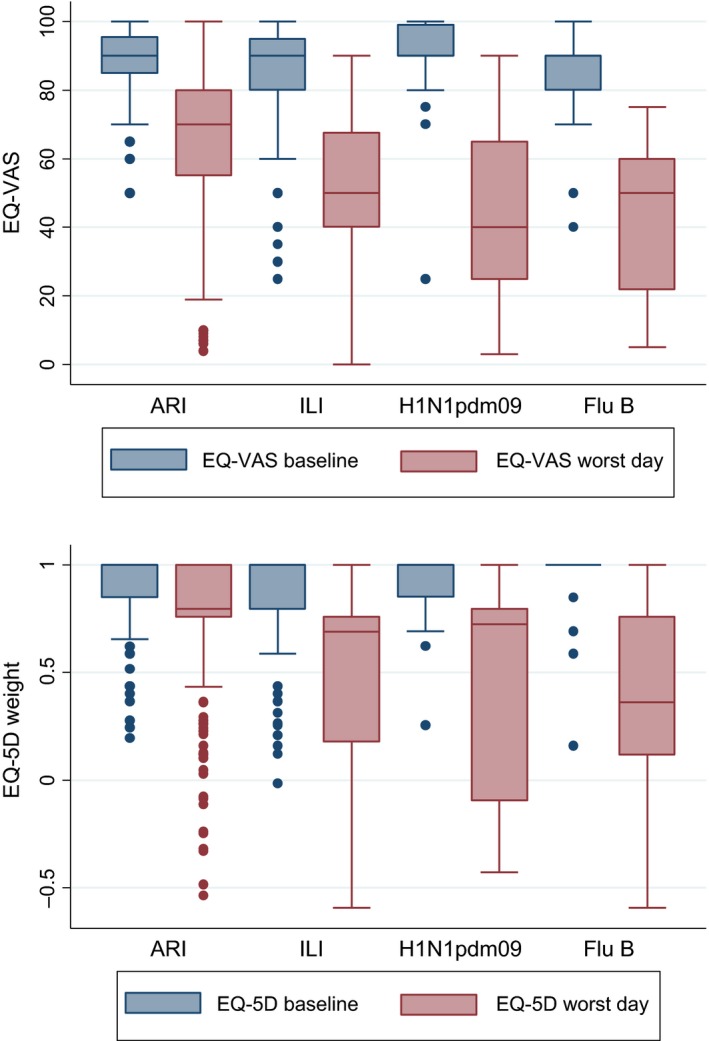
EQ‐VAS and EQ‐5D QALD weights comparing background and worst day of illness by illness outcome

The median and mean EQ‐VAS background scores were between 84 and 90 for H1N1pdm09, influenza B and ILI, but dropped to between 40‐50 on the worst day of illness (Figure 2, Table 3). Mean QALD weights were 0.93 and 0.92 at baseline for H1N1pdmo09 and influenza B, respectively, but dropped to 0.44 and 0.36 on the worst day of illness (Table 3). Median QALD weight for H1N1pdm09 (0.73) was much higher than the corresponding mean (0.44) suggesting that a few severe illnesses were greatly contributing to the mean (Figure 2, Table 3).

**Table 3:** Impact on health‐related quality of life (winter 2010/11)

	ARI		ILI		H1N1pdm09 PCR+		Flu B PCR+	
	N	Estimate	N	Estimate	N	Estimate	N	Estimate
Duration of symptoms, mean (min‐max, median)	413	7.5 (1‐42, 6.0)	256	9.9 (1‐65, 7.0)	35	8.8 (1‐26, 7.0)	23	11.9 (1‐65, 7.0)
VAS background, mean (min‐max, median)	408	89.0 (50‐100, 90.0)	248	85.0 (25‐100, 90.0)	34	89.8 (25‐100, 90.0)	22	84.1 (40‐100, 90.0)
VAS worst day of illness, mean (min‐max, median)	413	66 (4‐100, 70)	256	51 (0‐90, 50)	35	43 (3‐90, 40)	23	43 (5‐75, 50)
EQ‐5D weight background, mean (min‐max, median)	406	0.92 (0.20‐1.00, 1.00)	246	0.87 (‐0.02‐1.00, 1.00)	34	0.93 (0.25‐1.00, 1.00)	21	0.92 (0.16‐1.00, 1.00)
EQ‐5D weight day of illness, mean (min‐max, median)	413	0.77 (‐0.54‐1.00, 0.80)	256	0.48 (‐0.59‐1.00, 0.69)	35	0.44 (‐0.43‐1.00, 0.73)	23	0.36 (‐0.59‐1.00, 0.36)
QALDs lost, mean (min‐max, median)	405	0.26 (‐5.32‐11.47, 0.20)	246	0.93 (‐25.28‐14.48, 0.74)	34	1.61 (‐0.92‐6.66, 1.00)	21	1.84 (‐2.72‐10.83, 1.14)
By age group								
0‐15	84	0.24 (‐2.72‐7.22, 0.00)	71	0.20 (‐25.28‐4.65, 0.66)	7	1.08 (0.00‐4.27, 0.20)	10	1.82 (0.58‐3.27, 1.86)
16‐65	257	0.34 (‐5.32‐11.47, 0.20)	137	1.30 (‐4.72‐14.48, 0.82)	23	1.74 (‐0.92‐6.66, 1.15)	7	2.37 (‐0.84‐10.83, 1.02)
65+	64	‐0.03 (‐5.02‐3.37, 0.00)	38	0.99 (‐3.58‐7.96, 0.74)	4	1.75 (‐0.78‐5.47, 1.15)	4	0.95 (‐2.72‐3.15, 1.68)
QALYs lost, mean (min‐max, median)	405	0.0007 (‐0.0146‐0.0314, 0.0006)	246	0.0026 (‐0.0692‐0.0397, 0.0020)	34	0.0044 (‐0.0025‐0.0182, 0.0027)	21	0.0050 (‐0.0074‐0.0296, 0.0031)
By age group								
0‐15	84	0.0007 (‐0.0075‐0.0198, 0.0000)	71	0.0005 (‐0.0692‐0.0127, 0.0018)	7	0.0029 (0.0000‐0.0117, 0.0006)	10	0.0050 (0.0016‐0.0090, 0.0051)
16‐65	257	0.0009 (‐0.0146‐0.0314, 0.0006)	137	0.0035 (‐0.0129‐0.0397, 0.0022)	23	0.0048 (‐0.0025‐0.0182, 0.0032)	7	0.0065 (‐0.0023‐0.0296, 0.0028)
65+	64	‐0.0001 (‐0.0138‐0.0092, 0.0000)	38	0.0027 (‐0.0098‐0.0218, 0.0020)	4	0.0048 (‐0.0021‐0.0150, 0.0032)	4	0.0026 (‐0.0074‐0.0086, 0.0046)
QALDs lost (sensitivity analysis), mean (min‐max, median)	405	0.72 (0.00‐11.47, 0.41)	246	1.97 (0.00‐16.33, 1.15)	34	1.89 (0.00‐7.12, 1.09)	21	2.64 (0.00‐10.83, 1.46)
By age group								
0‐15	84	0.54 (0.00‐7.22, 0.20)	71	1.68 (0.00‐7.98, 1.03)	7	1.08 (0.00‐4.27, 0.20)	10	1.82 (0.58‐3.27, 1.86)
16‐65	257	0.77 (0.00‐11.47, 0.47)	137	2.02 (0.00‐14.48, 1.27)	23	1.98 (0.20‐6.66, 1.22)	7	2.80 (0.00‐10.83, 1.02)
65+	64	0.74 (0.00‐7.17, 0.41)	38	2.37 (0.00‐16.33, 1.04)	4	2.81 (0.19‐7.12, 1.97)	4	4.41 (0.84‐7.17, 4.81)
QALYs lost (sensitivity analysis), mean (min‐max, median)	405	0.0020 (0.0000‐0.0314, 0.0011)	246	0.0054 (0.0000‐0.0447, 0.0031)	34	0.0052 (0.0000‐0.0195, 0.0030)	21	0.0072 (0.0000‐0.0296, 0.0040)
By age group								
0‐15	84	0.0015 (0.0000‐0.0198, 0.0006)	71	0.0046 (0.0000‐0.0218, 0.0028)	7	0.0029 (0.0000‐0.0117, 0.0006)	10	0.0050 (0.0016‐0.0090, 0.0051)
16‐65	257	0.0021 (0.0000‐0.0314, 0.0013)	137	0.0055 (0.0000‐0.0397, 0.0035)	23	0.0054 (0.0006‐0.0182, 0.0034)	7	0.0077 (0.0000‐0.0296, 0.0028)
65+	64	0.0020 (0.0000‐0.0196, 0.0011)	38	0.0065 (0.0000‐0.0447, 0.0028)	4	0.0077 (0.0005‐0.0195, 0.0054)	4	0.0121 (0.0023‐0.0196, 0.0132)

**Figure 3:**
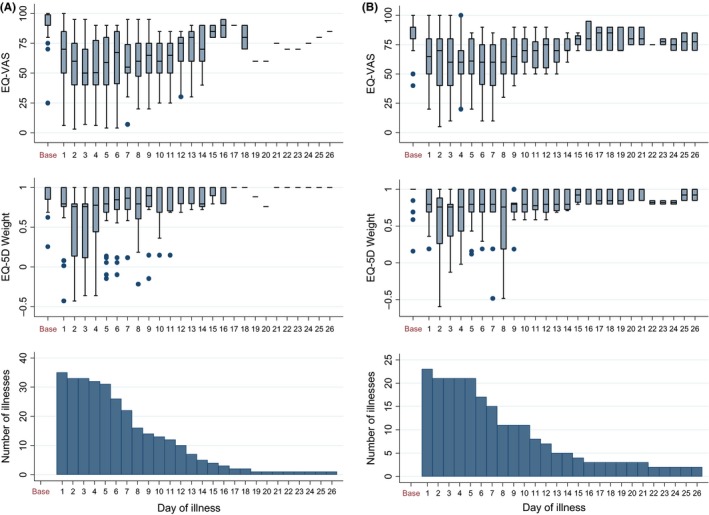
VAS and EQ‐5D‐3L QALD weight at baseline and by day of illness for (A) H1N1pdm09 illnesses and (B) Influenza B illnesses over the number of cases reporting symptoms on that day

For H1N1pdm09 and influenza B, daily EQ‐VAS and QALD weights varied throughout illness, with a rapid decline in the first 2 days (Figure 3a‐b). The lag time between symptom onset and the most severe day of illness appeared longer for H1N1pdm09 than for influenza B. Although the medians remain relatively low for the first week, over time these estimates reflected fewer illnesses, that is those with the longest duration (see bottom panels, Figure 3a‐b).

Average illness duration for H1N1pdm09 and influenza B cases with QALD data was 8.8 and 11.9 days, respectively, with 3% and 9% of illnesses, respectively, lasting over 3 weeks. Overall 1.61 QALDs were lost during H1N1pdm09 illnesses. QALD loss increased with age from 1.08 in children, to 1.74 and 1.75 in adults and the older adults, respectively. Influenza B illnesses lost more QALDs at 1.84 with age‐specific estimates of 1.82, 2.37 and 0.95 for children, adults and older adults, respectively. QALD loss during ILI and ARI illnesses was lower (0.26 and 0.93, respectively). Median QALD loss was typically lower than the mean for all illness outcomes, indicating that a small proportion of severe illnesses contributed greatly to the mean. 19% of H1N1pdm09 and 17% of influenza B cases with QALD/QALY data were medically attended. Mean QALD loss was 3.63 for medical‐attended H1N1pmd09 cases and 1.08 for non‐medically attended cases. Corresponding figures for influenza B were 5.48 and 1.23.

In sensitivity analysis, overall QALDs lost were higher at 1.89 for H1N1pdm09 and 2.64 for influenza B. Age‐specific sensitivity estimates were similar to the main analysis except in the oldest age group where the sensitivity analysis reports higher QALD losses.

### 3.3 Missing data

One H1N1pdm09 and 2 influenza B illnesses were missing baseline EQ‐5D‐3L measurements. Among the 57 influenza cases with QALD data, all but 2 reported no illness in the week following their illness.

### 3.4 Population impact

The estimated number of QALYs lost due to influenza A and B in England was 24 300 (95%CI: 16 600‐34 700), of which two‐thirds occurred in the 16‐64 years age group (Table 4). The estimated number of days off school in individuals aged 5‐15 years with influenza was 1.12 million (95%CI: 0.661‐1.78 million) per winter, of which 85% was associated with influenza A. The estimated number of days off work or education in individuals aged 16‐64 years with influenza was 1.79 million (95%CI: 1.16‐2.78 million), almost all of which (>98%) was due to influenza A.

**Table 4:** Population‐level burden of HRQoL lost and work/education absences due to community cases of influenza

	Age group	Flu type	Estimate	95% CI
QALY loss	Overall	A+B	24 300	16 600‐34 700
	By age group			
	0‐15	A+B	6410	3640‐10 900
	16‐64	A+B	16 200	9710‐25 800
	65+	A+B	1660	490‐4860
Days off work/education	Overall	A+B	2 910 000	2 090 000‐3 930 000
	By age group and flu type			
	5‐15	A	949 000	528 000‐1 580 000
		B	1 760 000	1 140 000‐2 610 000
	16‐65	A	170 000	52 300‐414 000
		B	27 600	4720‐89 100

## 4 DISCUSSION

### 4.1 Summary of results

We estimate that community cases of ARI, ILI, H1N1pdm09 and influenza B lose 0.25, 0.93, 1.61 and 1.84 QALDs from their illnesses, respectively. Our estimated QALDs lost increased with age which is consistent with previous findings.^8^ Mean QALD loss was much greater in medically attended H1N1pdm09 and influenza B cases (3.63 and 5.48, respectively) compared to non‐medically attended cases (1.08 and 1.23, respectively). We found 50% of influenza A illnesses and 41% of influenza B illness required someone (ill participant and/or their carer) to take time off work/education for a combined average of 5.0 and 3.4 days. Compared with adults, children with influenza were more likely to take time off education/childcare and to require someone else to take time off to care for them. Around a third of working adults required time off work for both influenza A and B illnesses with an average of 4 and 3 days off, respectively. Illness duration and time off estimates for ILI were comparable to influenza but higher than ARI. In England, community influenza cases lost 24 300 QALYs (8.87 million QALDs) in 2010/2011 and had an estimated 2.9 million absences per season based on data from 2006/2007 to 2009/2010.

### 4.2 Comparison with other studies

Previous studies show substantial variation in the HRQoL associated with influenza. This reflects differences in subjects' ages, definitions and severity of illness as well as the methods used to estimate HRQoL. Several estimates have been derived from cases seeking medical attention. In a population‐based study conducted in England during the 2009 pandemic using EQ‐5D‐3L, 2.92 QALDs were lost for confirmed cases of H1N1pdm09 and 2.74 for ILI controls.^5^ Another study reported a QALD loss of 1.68 for ILI due to confirmed influenza and 1.57 for non‐influenza ILI in adult patients.^9^ This was calculated by subtracting VAS scores presented by O'Brien et al^20^ from pooled oseltamivir trial data in nearly 640 ILI patients who received placebo, from a baseline quality of life weight. A study used data from the same trials to estimate the QALD loss associated with ILI as 5.33 in people aged 0‐19 years, 6.35 in people aged 20‐64 years and 10.69 for people aged 65 years and over by combining the published QALY weights with unpublished data on disease duration.^8^ Finally, a study of patients from hospitals and primary care centres with confirmed H1N1pdm09 in Spain showed individual QALD losses of 3.29 for primary care patients and 11.3 for hospitalised in‐patients.^11^

There are fewer studies of community influenza cases that may not consult healthcare professionals. Nevertheless, a survey in England of caregivers of children in primary school reporting ILI outbreaks that used EQ‐5D‐3L showed a mean loss of 2.1 QALDs.^1^ In Belgium, a household telephone survey including 2250 individuals with self‐reported ILI used SF‐12 to calculate QALDs lost: for an average episode of illness in the community, 1.83 QALDs were lost.^21^

In general, our estimates for individual‐level QALDs lost due to influenza were lower than earlier findings. This is unsurprising, as our study captured mild illnesses including cases of confirmed influenza that neither consulted for care nor met the symptom definition of ILI. Additionally, our study included children who typically have less severe disease as well as a large number H1N1pdm09 cases which in our cohort were less severe than H3N2 cases.^12^ This work and previous studies have shown that more QALDs are lost when estimates are derived from medically attended case, and in particular hospitalised cases. Our findings for work and school absences were also generally lower than previous estimates; for most illnesses, people did not take time off, although there were differences by age and illness definition. We showed however, that illness in a household member caused a substantial proportion of people take time off work to care for unwell household members. A study in the USA on school and parental absenteeism showed that for every 3 days a child took off school a parent missed on average 1 day of work.^22^

The aforementioned British and Spanish studies are not directly comparable as they estimated the population‐level burden of QALY loss due to influenza for more severe cases in a different season (2009/10).^5^, ^11^ They do however contextualise our findings as they report burden of QALY loss due to hospitalisations and deaths, which when combined with our results for community cases provides an indication of the scale of QALYs lost in a given season and the proportion attributable for different levels of disease severity. For example, the British study estimated that 40% (approximately 11 000 QALYs) of their total QALYs lost came from 337 reported influenza deaths.^5^ Similarly, the Spanish study estimated their 318 deaths lost 12 000 QALYs.^11^ It also estimated burden of QALY loss for influenza in‐patients and primary care patients, demonstrating that less severe yet more numerous primary care patients lost far more QALYs (6778) than the more severe but less common in‐patients (94 QALYs). Given these findings it seems that at least for these 2 seasons, the biggest contributors of population‐level QALY loss are community cases (medically and non‐medically attended) and deaths. The true burden and contribution by level of severity are likely to vary substantially between seasons and populations as it dependent on population size and age‐specific rates of illness and death. The estimated burden is also highly dependent on severity of cases included in the model.

### 4.3 Strengths and weaknesses

Our estimates of HRQoL and work and school absence were derived from a large community cohort study using active molecular and symptom surveillance to identify episodes of influenza, ILI and ARI. We captured a broad spectrum of illnesses including mild cases of laboratory‐confirmed influenza that did not meet the syndromic definition of ILI and/or did not consult a healthcare professional, which gave less biased estimates of the overall HRQoL and absences associated with influenza. A key strength was that participants completed the EQ‐5D‐3L daily over the course of an illness. This directly measures HRQoL throughout illness, so unlike other studies that used a single estimate of HRQoL during illness, we did not need to make assumptions about the shape of the QALY loss. The estimates for our population projections were all derived from the same data source.

Although we measured work and school absences over multiple years, HRQoL was only measured in 2010/11 when influenza A H1N1pdm09 and influenza B strains circulated. We expect that, as H3N2 was associated with more severe symptoms than H1N1, its effects on HRQoL might have been greater.^12^ Despite the large cohort size, the numbers with confirmed influenza and EQ‐5D were relatively low (N = 58) and not sufficient to draw conclusions on differences in HRQoL by strain. The uncertainty in our QALD and QALY estimates is reflected in the 95% confidence intervals of our population projections. We previously showed that most influenza infections are either asymptomatic or produce only mild illness.^12^ It is possible that we failed to capture very mild cases that did not shed enough virus to be PCR detectable. This would lead to a slight overestimation of individual‐level QALD loss associated with influenza illness. Conversely, our population‐level estimates should be considered minimum estimates because if we missed cases (eg from low viral shedding), this would reduce our estimated disease rates and thus overall burden estimates. We found some people reported worse HRQoL at baseline than during illness and our sensitivity analysis showed that when we took the participants' best reported measure of HRQoL as the comparison group, regardless of its timing, the oldest age group had much higher estimates of QALY loss. A further limitation is that children's HRQoL was reported by their parents. Previous studies show significant differences when both parents and adolescent measure children's quality of life.^23^ Instruments such as EQ‐5D‐3L have not been validated for use in infants and very young children, which is a challenge of assessing HRQoL in this age group.^24^

### 4.4 Implications

Estimates of QALDs lost and work and school absences associated with influenza differ depending on the setting in which cases are identified; community illnesses result in smaller effects but contribute substantially to the population‐level burden. Accurate assessment of both the number of expected cases and their QALDs/QALYs is essential to inform CUAs for decision‐making bodies such as NICE. While for some interventions, such as antiviral treatments of severe influenza cases, it is appropriate to use utility estimates derived from medically attended cases, we believe that our estimates are more appropriate for assessing cost utility of community preventive interventions such as vaccines.

## 5 CONCLUSIONS

We present new estimates of individual‐ and population‐level QALDs and QALYs lost and work and school absences due to community cases of influenza to inform CUAs of community interventions to prevent influenza.

## COMPETING INTERESTS

EBF, CWG, MZ and PJW report no conflict of interests. ACH serves on the Department of Health for England Joint Committee for Vaccination and Immunisation Influenza Subgroup and the Department of Health for England New and Emerging Respiratory Virus Threats Advisory Group. WJE's partner works for GlaxoSmithKline, who manufacture influenza vaccines and antivirals. JSN‐V‐T reports research grants, unrelated to the present work from F. Hoffmann‐La Roche and GlaxoSmithKline; consultancy for Prep Biopharm, and ACM Biolabs; and a travel grant from the European Scientific Working Group on Influenza (ESWI) to enable delivery of a plenary lecture.
